# Zoster Sine Herpete Masquerading as Central Nervous System Vasculitis

**DOI:** 10.7759/cureus.7231

**Published:** 2020-03-10

**Authors:** Arthur Lau, Eno-Obong Essien, Irene J Tan

**Affiliations:** 1 Rheumatology, Temple University Hospital, Philadelphia, USA; 2 Internal Medicine, Temple University Hospital, Philadelphia, USA; 3 Rheumatology, Einstein Medical Center, Philadelphia, USA

**Keywords:** vasculopathy, varicella zoster virus, cns vasculitis, immunocompromised, vasculitis, zoster sine herpete, shingles, hiv

## Abstract

Central nervous system (CNS) vasculopathy caused by varicella zoster virus (VZV) is a rare condition. Rarer still is the development of CNS vasculopathy in the absence of a typical zoster rash, a phenomenon known as zoster sine herpete. We report a case of a 34-year-old male with HIV, non-compliant with highly active antiretroviral therapy (HAART), who presented with left-sided temporal headaches and numbness without rash. The patient had a complicated one-month hospital stay when he was initially diagnosed with mycobacterium avium complex (MAC) tuberculosis infection and treated with isoniazid, rifabutin, ethambutol, and azithromycin. Additionally, he was thought to have immune reconstitution inflammatory syndrome (IRIS) and was given steroids. Unfortunately, he presented one day post-discharge with lethargy, aphasia, and dysphagia and was found to have acute/subacute infarcts affecting multiple areas of the brain. CT angiogram (CTA) of the brain showed evidence of multifocal areas of mild to moderate stenosis throughout the intracranial arterial circulation. The patient underwent conventional angiography, which showed segmental arterial constrictions with post-stenotic dilatation consistent with vasculitis. Cerebrospinal fluid (CSF) studies eventually returned positive for VZV by polymerase chain reaction (PCR), confirming a diagnosis of VZV-induced CNS vasculopathy, or more specifically, CNS vasculopathy due to zoster sine herpete. The patient was treated with high-dose steroids as well as IV acyclovir with improvement in his symptoms. He was discharged with advice for a close follow-up with the infectious disease (ID) department. Our case highlights the importance of maintaining a high index of suspicion for varicella infection masquerading as CNS vasculitis, particularly in the absence of classic blistering shingles rash. Early detection may prevent neurological sequelae of the infection, including stroke, dissection, or neuropathy.

## Introduction

Varicella zoster virus (VZV) vasculopathy is a rare disease affecting the central nervous system (CNS). It is characterized by VZV infection of arteries, which may lead to vasculitis resulting in transmural inflammation and vessel wall damage [1]. Recognizing VZV vasculopathy early is important as a delay in treatment may lead to complications including ischemic or hemorrhagic stroke, aneurysm formation, dissection, cranial neuropathies, and myelopathy among others [2]. All of these complications of varicella infection may occur in the absence of a rash (zoster sine herpete), making it diagnostically challenging to identify the condition [3]. We report a rare presentation of VZV vasculopathy masquerading as CNS vasculitis in an immunocompromised male with HIV who presented with zoster sine herpete.

## Case presentation

**Figure 1:**
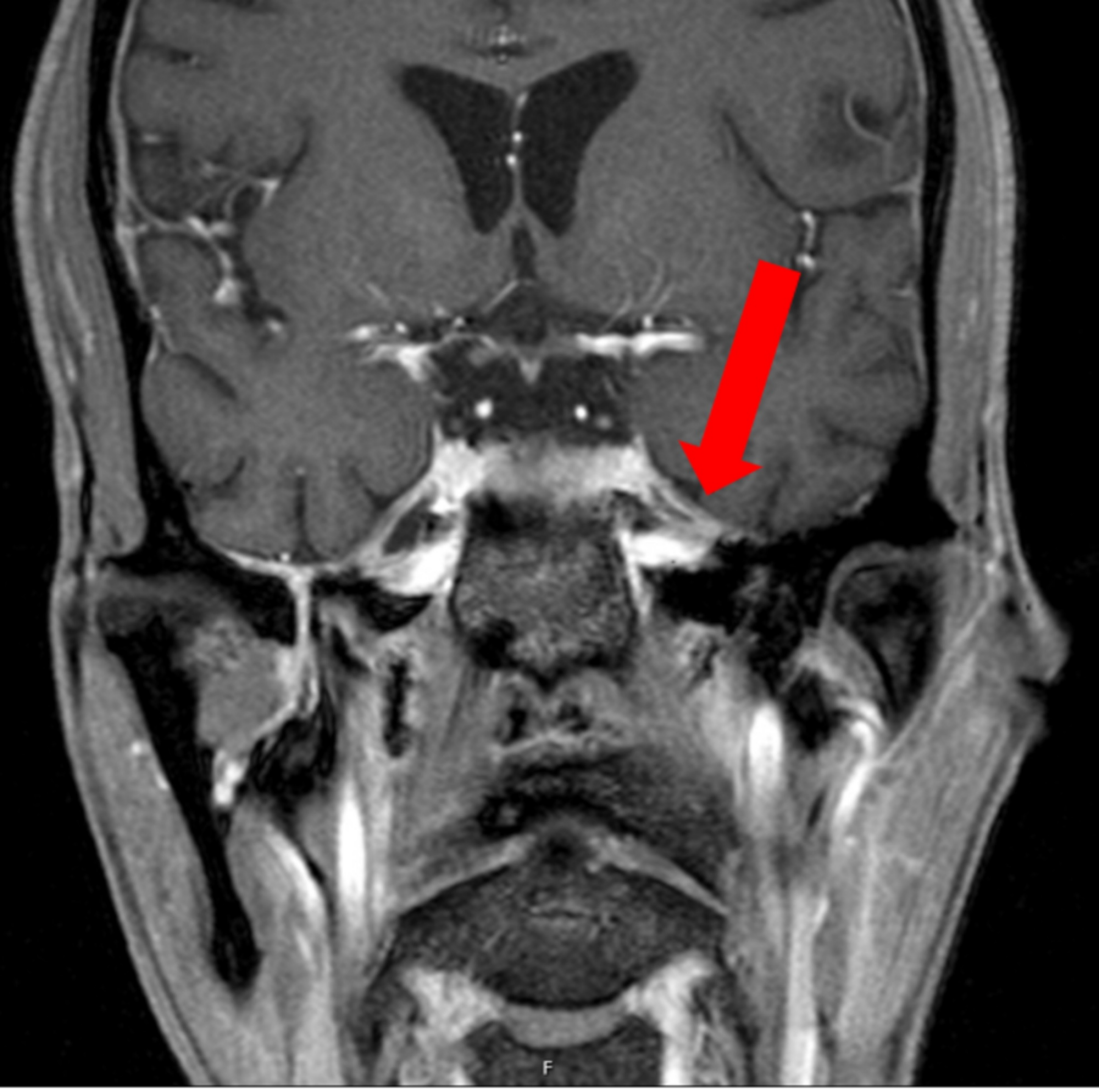
MRI of the brain with contrast The image shows focal meningeal thickening compressing the cistern of the left trigeminal ganglion (red arrow) MRI: magnetic resonance imaging

A 34-year-old male with a past medical history of HIV and non-compliance with highly active antiretroviral therapy (HAART), with a last known CD4 count of 45, presented to the hospital with a left-sided temporal headache with associated numbness, decreased visual acuity, and scleral injection of the left eye. He also reported weight loss, poor appetite, and night sweats of a one-month duration with associated diurnal fevers. Physical exam revealed a cachectic-appearing male with thrush in his mouth, no rashes, and decreased sensation in the V1 distribution of the left side of his face. The patient underwent a lumbar puncture, which showed a white blood cell (WBC) count of 6 K/uL (96% lymphocytes, 1% neutrophils), red blood cell (RBC) count of 19 K/uL, glucose level of 36 mg/dL, and protein of 47 mg/dL [cerebrospinal fluid (CSF) studies were not performed on this admission]. CT and MRI of his brain without contrast were unremarkable. He was evaluated by ophthalmology who noted a corneal abrasion affecting his left eye, which was attributed to HIV retinopathy. Given the initial concern for meningitis, he was empirically placed on antibiotics and IV acyclovir by infectious diseases (ID) consultant for five days before they were discontinued. Neurology was consulted given persistent left temporal numbness, but they were unsure of the underlying etiology. He underwent a magnetic resonance angiography (MRA) of the head and neck with and without contrast, which returned normal. He then had an MRI of his brain with contrast, which showed focal meningeal thickening compressing the cistern of the left trigeminal ganglion with questionable enhancement extending into the pterygopalatine fossa and inferior orbital foramen (Figure 1).

**Figure 2:**
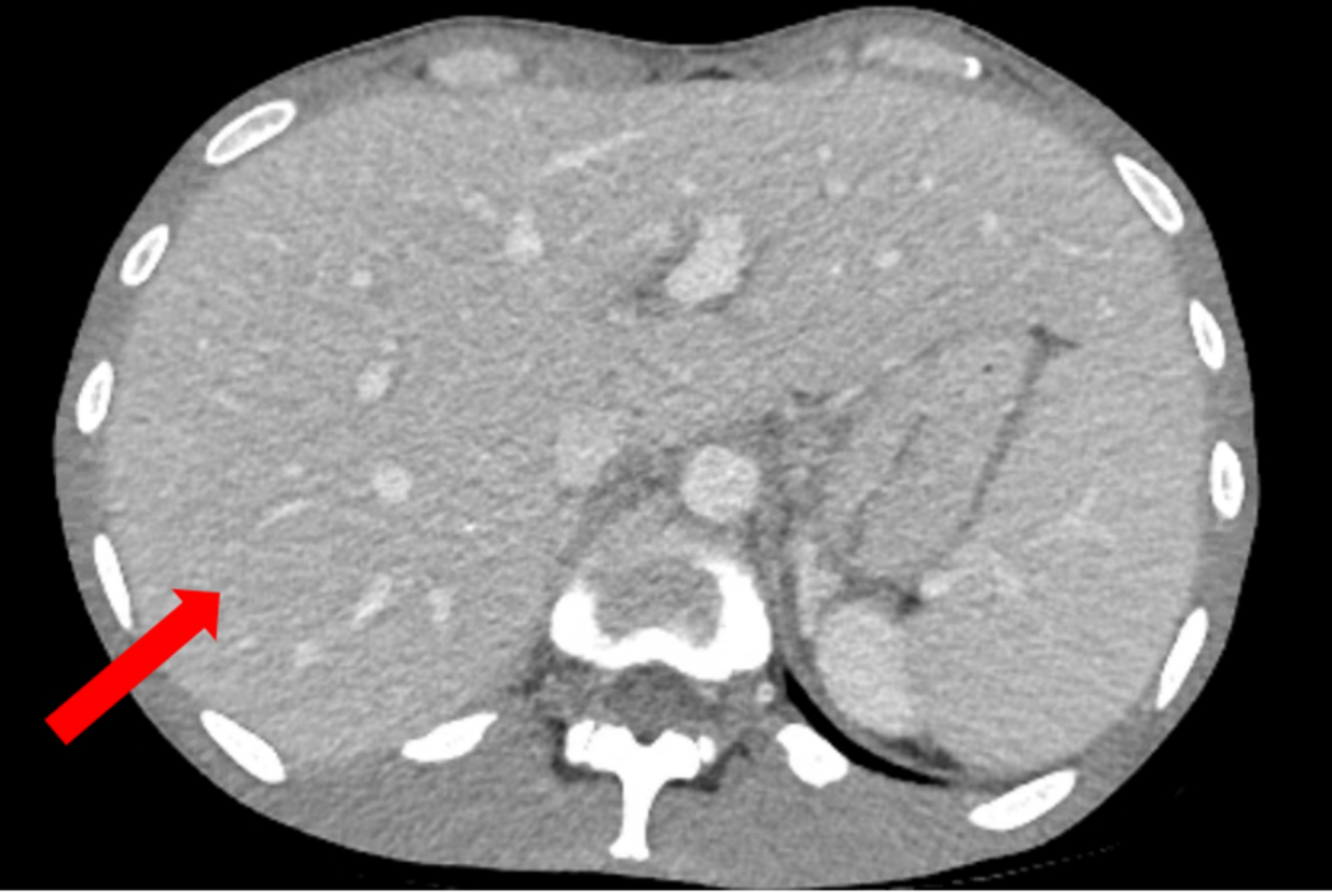
CT of the abdomen with IV contrast The image shows hepatomegaly (red arrow) CT: computed tomography; IV: intravenous

He also had a CT of the chest/abdomen/pelvis, which showed hepatomegaly and extensive retroperitoneal, periaortic, and mesenteric adenopathy concerning for lymphoma (Figure 2).

He ultimately underwent a liver biopsy, which showed multiple caseating granulomas, and lymph node biopsy, which was nonspecific but not consistent with malignancy. He was empirically started on therapy for mycobacterium avium complex (MAC) tuberculosis infection with isoniazid, rifabutin, ethambutol, and azithromycin. MAC eventually grew from liver biopsy culture, confirming the diagnosis. He was subsequently started on steroid therapy with methylprednisolone 40 mg IV twice a day for a couple of days given concern for immune reconstitution inflammatory syndrome (IRIS) in the setting of newly restarting HAART therapy and with the treatment of AIDS-defining illness. He was discharged on treatment for MAC and was prescribed prednisone 20 mg oral daily. The patient’s symptoms, including the numbness of his face, improved and he was discharged after a month-long hospital stay with advice for close follow-up.

**Figure 3:**
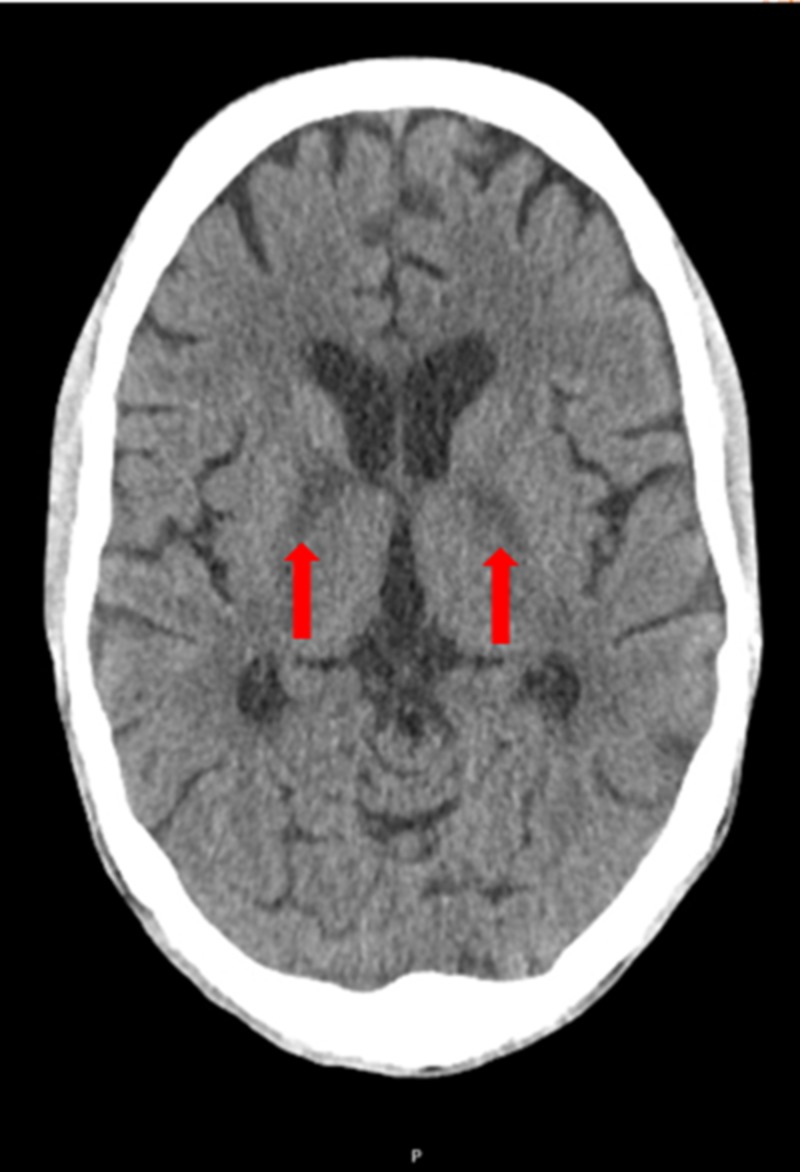
CT of the brain without contrast The image shows new symmetric hyperdensities (red arrows) CT: computed tomography

One day post-discharge, the patient’s wife observed that he had developed new-onset aphasia, global weakness, and difficulty swallowing. He was subsequently brought back to the hospital. Physical examination was significant for a minimally verbal and lethargic male. His cranial nerve exam was intact. There was a mild diminution of strength in the bilateral lower extremities, negative Romberg, no clonus, and down-going plantar reflex. The skin exam revealed no petechiae, bruising, or rash. He was empirically started on IV acyclovir at this time. Blood tests with normal or negative results included vitamin B1, B12, methylmalonic acid, ammonia, syphilis rapid plasma reagin cascade, urine drug screen, and urinalysis. Rheumatologic workup, which included antinuclear antibody (ANA), antineutrophil cytoplasmic antibodies (ANCA), rheumatoid factor (RF), C3, C4, anti-Sjögren’s-syndrome-related antigens A and B (anti-SSA and SSB), and antiphospholipid antibodies, were all unremarkable. Erythrocyte sedimentation rate (ESR) and C-reactive protein (CRP) were elevated. Lumbar puncture was performed again, which showed a WBC of 2 K/uL (70% lymphocytes, 5% neutrophils), RBC of 0 K/uL, a glucose level of 40 mg/dL, and protein of 68 mg/dL. CSF studies including cryptococcal antigen, syphilis, herpes simplex virus, BK virus, John Cunningham (JC) virus, VZV, acid-fast bacillus (AFB)/fungal and bacterial cultures were sent. The patient underwent a CT of his head without contrast, which showed new symmetric hyperdensities along the bilateral internal capsules and possibly bilateral globus pallidus (Figure 3).

**Figure 4:**
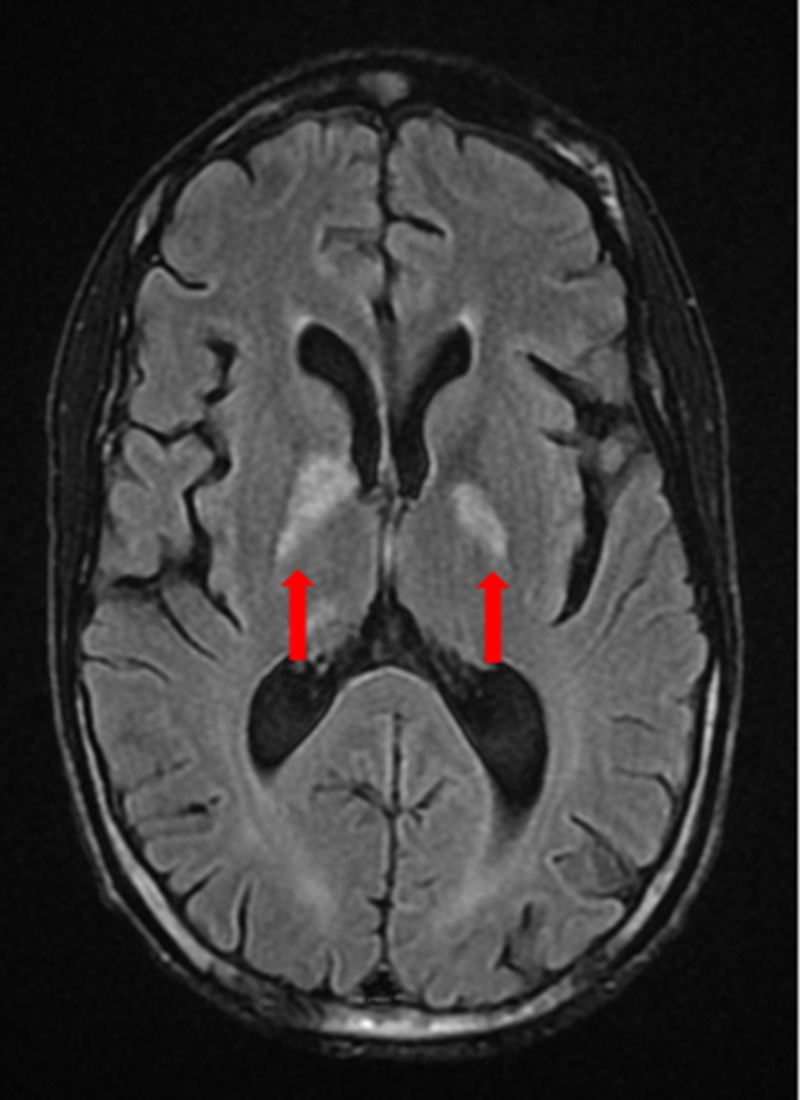
MRI of the brain with and without contrast The image shows acute/subacute infarcts (red arrows) MRI: magnetic resonance imaging

Following a neurology consult, he had an MRI of the brain with and without contrast, which showed acute/subacute infarcts involving the bilateral globus pallidus, bilateral posterior limbs of the internal capsule, and smaller scattered infarcts in anterior and posterior circulation distribution (Figure 4).

**Figure 5:**
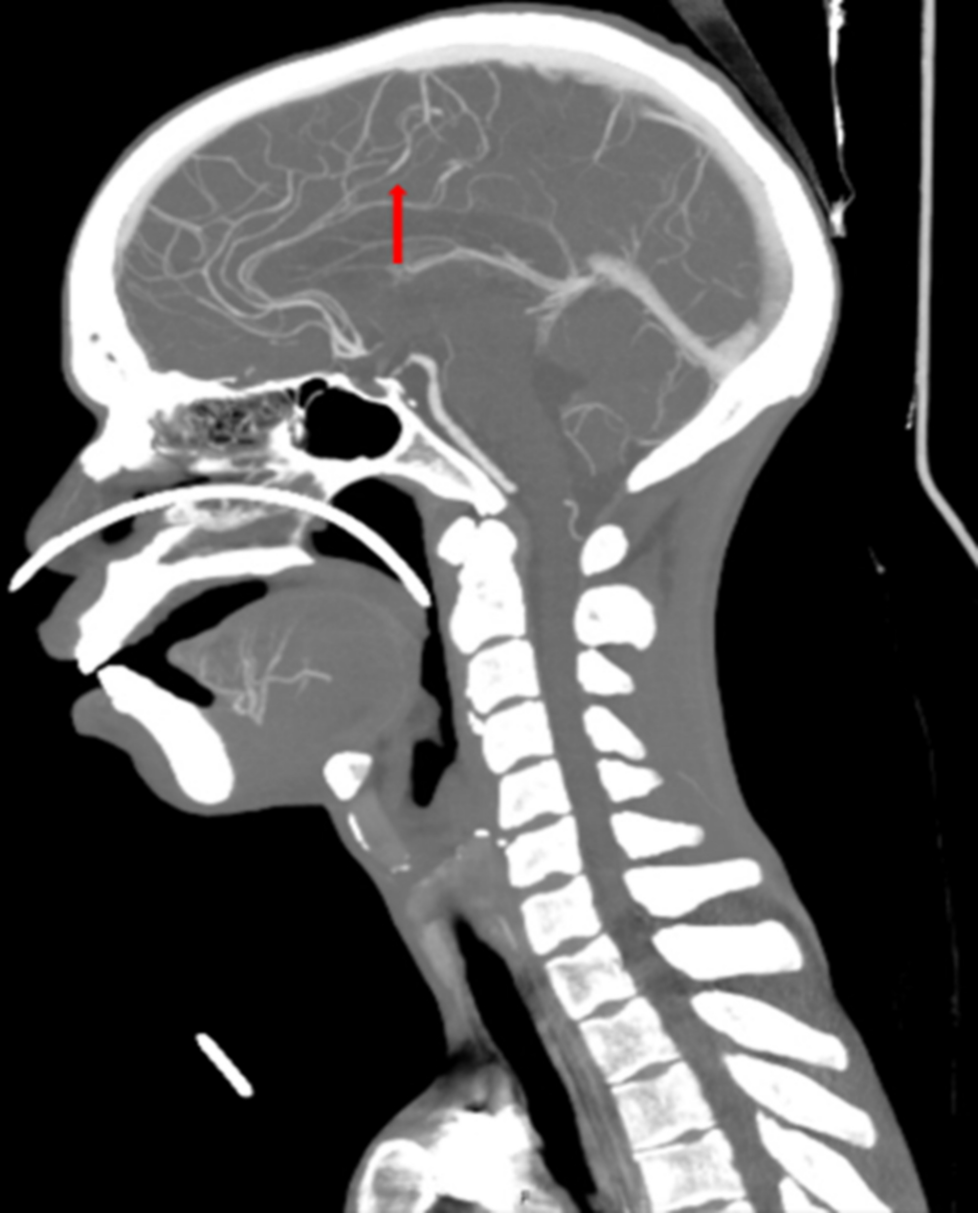
CTA of the head and neck The image shows multifocal areas of mild to moderate stenosis (red arrow) CTA: computed tomography angiography

The patient also had a CT angiography (CTA) of the head and neck, which demonstrated multifocal areas of mild to moderate stenosis throughout the intracranial arterial circulation, predominantly involving the second- and third-order branches (Figure 5).

**Figure 6:**
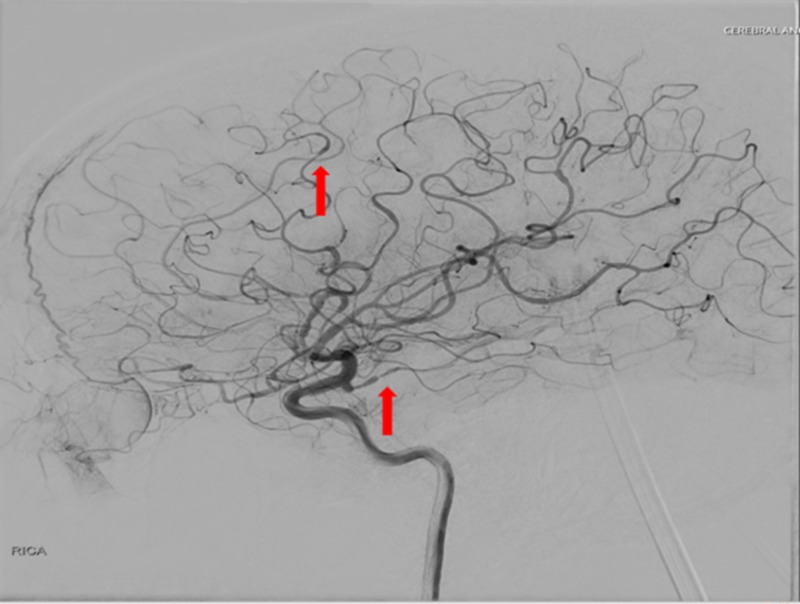
Conventional cerebral angiography The image demonstrates areas of stenosis and ectasia (red arrows)

To rule out other vessel involvement, CTA of the thorax, abdomen, and pelvis were also performed and were found negative for vasculitis. A neurosurgical consultation was obtained and conventional cerebral angiography was performed, demonstrating areas of stenosis and ectasia in the bilateral middle cerebral artery, anterior cerebral artery, posterior communicating artery, distal basilar artery, and proximal posterior cerebral artery, consistent with CNS vasculitis (Figure 6).

Additionally, as part of the stroke workup, a transthoracic echocardiogram was done, which showed a decreased ejection fraction of 20-25% percent with regional wall motion abnormalities, but no evidence of thrombus. Cardiology attributed the changes to HIV cardiomyopathy.

Given the results of the imaging findings, an interdisciplinary meeting of rheumatology, neurology, and ID services agreed on a management plan to treat the presumed primary CNS vasculitis. He was empirically treated with dexamethasone 110 mg daily IV for three days and rituximab 500 mg IV once. Brain biopsy was deferred given the high-risk nature of the procedure and the patient’s immunocompromised status. The patient’s mental status and neurologic exam showed improvement after the initiation of therapy. On hospital day nine, or four days after initiation of steroids and one dose of rituximab, the patient’s CSF returned positive for VZV DNA by PCR. The presumptive CNS vasculitis was now confirmed to be VZV-mediated and he was diagnosed as having VZV, or more specifically, zoster sine herpete-induced CNS vasculopathy. Steroids and acyclovir were continued and rituximab was held. He continued to improve with the treatment. He was subsequently discharged alert, oriented, and conversant, to a skilled nursing facility with advice for close follow-up with the ID team.

## Discussion

VZV is an enveloped, spherical double-stranded DNA virus, and one of eight known human herpesviruses [4]. Primary infection with varicella virus usually occurs via aerosol from skin vesicles from an infected person with varicella, usually leading to varicella infection (chickenpox). VZV becomes latent after the primary infection within neurons in the cranial nerves, dorsal root, and autonomic ganglia. Latent VZV may be reactivated due to a decline in immunity in patients who are older or in patients who are immunocompromised. This reactivation leads to zoster (shingles) infection and may lead to other complications including vasculopathy and postherpetic neuralgia [5]. Notably, varicella has been shown to replicate in arteries leading to vasculitis, vessel wall damage, and transmural inflammation [1]. VZV vasculopathy may result in clinical features of stroke, aneurysm formation, myelopathy, cranial neuropathy, and dissection among other complications [2]. Significantly, VZV infection may lead to all of these complications despite the absence of a rash, a phenomenon known as zoster sine herpete, making it challenging to diagnose [3,6]. This is evidenced by our case, as VZV infection was not suspected during the patient’s first admission given his lack of characteristic rash.

In one of the largest case series of 30 patients with virologically confirmed VZV vasculopathy, rash occurred in 19 patients (63%), CSF pleocytosis in 20 patients (67%), and imaging abnormalities in 29 (97%). The CSF of only 9 patients (30%) contained VZV DNA, whereas 28 patients (93%) had an anti-VZV IgG antibody in the CSF [3,7]. This suggests that the diagnosis of VZV vasculopathy and its complications are often missed due to lack of zoster rash in about a third of the patients, and a normal CSF in a third of the patients. Even more daunting is that the average time of transition from zoster infection to neurologic symptoms and signs may be as long as four months [7]. Diagnosis can be made clinically based on characteristic rash and associated symptoms, but it is usually primarily based on virologic confirmation with IgM/IgG antibody in the CSF or VZV DNA in the CSF by PCR. VZV IgG persists after VZV PCR becomes undetectable, so a negative VZV PCR does not exclude VZV vasculopathy. There is also often mononuclear pleocytosis of <100 cells in the CSF and/or evidence of RBC [8]. Imaging studies may reveal infarcts often at the gray-white matter junction, vessel wall enhancement, or segmental stenosis with post-stenotic dilatation among others [8]. Pathology of infected arteries may reveal evidence of Cowdry A inclusion bodies, multinucleated giant cells, herpes virions, and both VZV DNA and antigen [9]. Unfortunately, there are no controlled trials to assess the optimal treatment strategy in the setting of VZV vasculopathy and treatment is often based on case reports and small studies. It is suggested that for suspected VZV vasculopathy, empiric IV acyclovir 10 mg/kg administered every eight hours should be initiated while waiting for confirmatory CSF testing. For patients with a confirmed diagnosis of VZV vasculopathy, acyclovir should be administered for a minimum of 14 days, and it should be extended for those who do not respond. Oral prednisone at a dosage of 1 mg/kg for 5 days without a taper is also given to decrease the inflammatory response in the cerebral arteries [5]. Vaccination should also be considered in the appropriate population, particularly with the advent of the new varicella zoster recombinant, adjuvanted vaccine (Shingrix, GlaxoSmithKline plc, Brentford, UK), to prevent the sequelae of varicella infection [10].

## Conclusions

We reported a rare presentation of VZV-induced CNS vasculopathy in the absence of a rash, a phenomenon known as zoster sine herpete. It is now known that VZV reactivation without a rash may lead to various neurological disorders associated with VZV, including CNS vasculopathy, in as high as one-third of the cases. As such, it is critical for clinicians to maintain a high index suspicion for VZV infection in the right clinical context, particularly in immunocompromised patients.
